# Application of ZnO Nanoparticles Phycosynthesized with *Ulva fasciata* Extract for Preserving Peeled Shrimp Quality

**DOI:** 10.3390/nano11020385

**Published:** 2021-02-03

**Authors:** Mohammed S. Alsaggaf, Amany M. Diab, Basant E.F. ElSaied, Ahmed A. Tayel, Shaaban H. Moussa

**Affiliations:** 1 Department of Biology, College of Science and Humanitarian Studies, Shaqra University, Qwaieah 11971, Saudi Arabia; dr.alsaggaf10@gmail.com (M.S.A.); shaus2008@yahoo.com (S.H.M.); 2 Department of Aquaculture, Faculty of Aquatic and Fisheries Sciences, Kafrelsheikh University, Kafrelsheikh 33516, Egypt; amany_diab@fsh.kfs.edu.eg; 3 Department of Fish Processing and Biotechnology, Faculty of Aquatic and Fisheries Sciences, Kafrelsheikh University, Kafrelsheikh 33516, Egypt; basantelsa3ed@gmail.com; 4 Department of Microbial Biotechnology, Genetic Engineering and Biotechnology Research Institute, University of Sadat City, El-Sadat City 22857, Egypt

**Keywords:** antimicrobial, foodborne pathogens, green synthesis, macroalgae extract, nano-metals, nanocomposite, shrimp

## Abstract

Zinc oxide nanoparticles (ZnONPs) were the targets of numerous biological syntheses to attain their precious values in various biomedical fields. The phycosynthesis of ZnONPs were innovatively investigated using cell-free extract of the macroalgae, *Ulva fasciata Delile*. The phycosynthesized *U. fasciata*-zinc oxide nanoparticles (UFD-ZnONPs) had 77.81 nm mean size, with flower and sphere shapes and positive zeta potential. The UFD-ZnONPs infra-red analysis indicated their basic components’ cross-linkage. The antibacterial potentialities of UFD-ZnONPs were confirmed, qualitatively and quantitatively, against foodborne microorganisms (*Escherichia coli* plus *Staphylococcus aureus*); the bactericidal action was higher for UFD-ZnONPs than the annealed phycosynthesized ZnONPs. The scanning micrographs of *S. aureus* and *E. coli* cells treated with UFD-ZnONPs indicated the severe action of nanoparticles to destroy bacterial cells in time-dependent manners. Peeled shrimps (*Fenneropenaeus indicus*) were biopreservated through refrigerated storage (4 °C) with UFD-ZnONPs based solution for six days. The microbial examination of UFD-ZnONPs -treated shrimps displayed decrease in microbial loads throughout the storage days. Moreover, the UFD-ZnONPs-treated shrimps showed acceptable sensorial attributes (appearance, odor, color and texture) compared to untreated shrimps. UFD-ZnONPs nanocomposite concentration of 3% and 5% could be remarkably suggested as efficient procedure for shrimps’ biopreservation during refrigerated storage regarding sensorial quality and microbial profile of product.

## 1. Introduction

Nanoparticles (NPs) frequently have sizes diameter of <100 nm/particle [1] and can have high advantages in almost all biological fields [2]. The accustomed methods to NP synthesis, e.g., physical and chemical protocols, possess many drawbacks, both economically and environmentally [3,4].

Biosynthesis is an ecofriendly, easy, and economical method that exploits living creatures, such as actinomycetes, algae, bacteria, viruses, fungi, and yeast, for NP production. Algae are known as “bio-nano-factories” because algal biomasses (live/dead) and their extracts are employed to phycosynthesize metallic and non-metallic NPs [5]. In macroalgae, the phycosynthesis has been mediated by a plurality of compounds, e.g., amines, amides, alkaloids, terpenoids, pigments, phenolics, proteins, etc., existing in the crude extracts, which helps in metal stabilization and reduction [6]. *Ulva fasciata Delile* is a green marine macroalga belonging to family Ulvaceae which is widely available on the north coast of Egypt. Recently, *U. fasciata* and its phenol-rich extract have been used to synthesize only silver NPs [7,8].

Zinc is a broadly existing essential element in various body tissues, e.g., muscles, brain, skin, and bones. As a key component of numerous enzyme systems, zinc plays vital roles in the body’s metabolism, protein and nucleic acid synthesis, neurogenesis, and hematopoiesis. Additionally, ZnO is cost-effective and non-toxic with safety recognition (GRAS) by the FDA (Food and Drug Administration) [9]. Among the metallic NPs, nano-zinc oxide particles (ZnONPs) have attracted extensive attention owing to these NP’s multifunctional nature. Principally, ZnONPs are generally employed in bio-imaging, sunscreens, UV photodetector, drug delivery, semiconductor diodes, ointments, and lotions due to their anticancer and antibacterial potentialities [10]. The ZnONPs with many shapes and sizes can be biologically manufactured from zinc salts, e.g., zinc acetate (Zn(CH_3_CO_2_)_2_), in the presence of reducing agents, such as phenols, flavonoids, and steroids [11]. ZnONPs have been biologically synthesized by different bacteria, like *Lactobacillus plantarum* [12], and plants, like the leaf extracts of *Deverra tortuosa* [13].

The conventional protocols for metal NP synthesis, e.g., *ZnONPs*, may have some advantages like time saving and large production potentiality, but they require high concerns regarding energy consumption and disposal of unsafe reducing chemicals [14]. Promising investigations indicated the capability of aquatic organisms, micro- and macro-algae materials, as “bio-nano-factories” for synthesizing such NPs [5,14,15].

Algal extracts are wealthy with various bioactive molecules, e.g., antioxidants (tocopherols, polyphenols), pigments like chlorophylls, phycobilins (phycoerythrin, phycocyanin), and carotenoids (carotene, xanthophyll) [14]. From available reports, these bioactive combinations were authenticated as potential reducing/stabilizing agents [5,8,14]. It has been recorded that *U. fasciata* extracts mainly contain phenolic compounds, where benzoic acid and gallic acid are the main ones, in addition to fatty acids and nonpolar compounds [5].

The fights against bacterial pathogens, especially from foodborne species (e.g., *Salmonella* spp., *Staphylococcus aureus*, *E. coli*, etc.), have high health concerns to control the disastrous consequences of these organisms that grow in most foodstuffs [16]. Aquatic environments have treasures of valuable products, mainly in the healthcare, medicinal, and nutraceutical segments [6,14]. Shrimps are highly perishable and can be attacked by foodborne pathogens, which cause hazardous outbreaks and diseases that could lead to death. Hence, the plan of this research was the innovative facile phycosynthesis of ZnONPs using algal sources and to characterize their physiognomies and evaluate their antibacterial potentiality against Gram-positive and Gram-negative foodborne pathogens and their application for peeled shrimps’ biopreservation.

## 2. Materials and Methods

### 2.1. Collection of Algal Material and Extract Preparation from Ulva fasciata Delile

Fresh marine macroalga, *U. fasciata* was attained from the NIOF (National Institute for Oceanography and Fisheries), Alexandria, Egypt. According to *Ishwarya* et al. [17], 5 *g* of *U. fasciata* dried powder was boiled in 50 mL double distillated water (dDW) for 20 min. *U. fasciata* extract was vacuum filtrated then centrifuged at 6430× *g* for 10 min. The resulting extract was vacuum evaporated at 42 °C then the dried powder was employed in further experiments.

### 2.2. Phycosynthesis of Ulva fasciata Delile-Zinc Oxide Nanoparticles (UFD-ZnONPs)

Sterilized MilliQ water (MQW) was applied for experiments solution preparation. A glass vial containing 10 mL of 10 mM of dihydrated zinc acetate “Zn(CH_3_CO_2_)_2_·2H_2_O” (Sigma–Aldrich, St. Louis, MO, USA) solution was stirred via a magnetic stirrer for 30 min. Drop-wise addition of freshly-prepared *U. fasciata* extract solution (2 mL, 1.0% concentration, *w*/*v*) was made in the vial. The mixture was stirred for 3–4 h at 72 °C until the solution color changed from green to pale white, indicating formation of phycosynthesized UFD-ZnONPs. Afterward, centrifugation of the reaction mixture (at 4100 rpm for 12 min) was conducted; the whitish sediments were gathered and washed with dDW. Pure ZnONPs were acquired via calcinating the UFD-ZnONPs mixture for 4 h at 455 °C in a muffle furnace.

### 2.3. Characteristics of the Phycosynthesized UFD-ZnONPs

#### 2.3.1. FTIR Analysis

Briefly, *U. fasciata* extract and UFD-ZnONPs solution was dried and ground into a homogeneous powder, and spectra were achieved at 500–4000 cm^−1^ wave numbers against potassium bromide (KBr) using a spectrophotometer (JASCO spectrometer 4100, Tokyo, Japan). The transmittance spectral peaks were then plotted.

#### 2.3.2. XRD Analysis

XRD measurements were made for pure ZnONPs using a diffractometer (XRD-6000, Shimadzu, Japan) with *λ* = 1.5412 Å Cu-*k_α_* radiation, within 2θ range of 10–80° at 30 mA and 40 Kv, for analysis of purity.

#### 2.3.3. EDX Analysis

The elemental analysis of pure ZnONPs was executed via EDX spectroscopy (JSM-IT100, JOEL, Tokyo, Japan).

#### 2.3.4. SEM Analysis

The sample surface topography of pure ZnONPs was studied by SEM (SEM-IT100, JEOL, Tokyo, Japan). The powder was finely dried into powder form by using a spray dryer. The powdered sample was carefully mounted onto stubs and placed into the sputter cortex chamber and covered with gold/palladium for SEM investigation to determine the structure of developed nanoparticles.

#### 2.3.5. Particle Size (Ps) Distribution and Zeta Potential (ζ) Analysis

The surface charges of the phycosynthesized UFD-ZnONPs were determined by their *ζ* potential and the Ps distributions of ZnONPs were determined via the DLS (dynamic light scattering) technique (Zeta plus, Brookhaven, NY, USA).

### 2.4. Evaluation of Antibacterial Potentiality

The antibacterial potentialities of *U. fasciata* extract and UFD-ZnONPs were evaluated, qualitatively and quantitatively, against the challenged bacterial strains.

#### 2.4.1. Challenged Bacterial Culture

*Staphylococcus aureus* (strain ID: ATCC 25923) and *Escherichia coli* (strain ID: ATCC 25922) bacterial strains were used as challenged models. The cultures were propagated and examined in Nutrient broth and agar (NB and NA) media (Himedia, Mumbai, India), temperature 37 ± 1 °C.

#### 2.4.2. Qualitative Antibacterial Potentiality: Inhibition Zone (IZ) Assay

The qualitative assay (using disc diffusion method), was mostly applied in dark to exclude the potential light effect on NPs activity. Bacterial cultures (24 h old) were spread onto NA dishes then sterile discs (6 mm diameter from Whatman filter paper, no. 1) were loaded with 30 µL of *U. fasciata* extract or UFD-ZnONPs solutions (each with 100 µg/mL concentration) and positioned on the inoculants surfaces. After incubation (for 24 h at 37 °C), the emerged IZ diameters were measured, and mean triplicate diameters were calculated.

#### 2.4.3. Quantitative Antibacterial Potentiality: Minimum Concentration for Inhibition (MIC)

The described microdilution technique of Tayel et al. [18] was employed to determine the MICs of *U. fasciata* extract or UFD-ZnONPs against examined foodborne bacteria. In 96-wells microplates, the bacterial cultures (~2 × 10^7^ CFU/mL) were challenged with serial concentrations of inspected agents (in the range of 1–200 µg/mL) then microplates were incubated as abovementioned and the cells viability were assessed using chromogenic indicator *p*-iodonitrotetrazolium violet aqueous solution (4% *w*/*v*, Sigma-Aldrich, St. Louis, MO, USA), which produces a red formazan color by active biological cells. Portions of wells containing inhibited cells were plated onto fresh NA plates and incubated to confirm the inhibitory action. The MIC was specified as the least concentration that prevented bacterial growth in microplates and on NA plates.

#### 2.4.4. Scanning Electron Microscopic (SEM) Imaging

The SEM imaging was employed to detect morphological alterations in *S. aureus* and *E. coli* cells, after exposure to UFD-ZnONPs, for potential elucidation of NPs action mode. The SEM (SEM-IT100, JEOL, Tokyo, Japan) bacterial imaging was conducted using the Marrie and Costerton protocol [19]. Grown bacterial cells in NB for 24 h were treated with UFD-ZnONPs (45 µg/mL) for 0 (control), 1 h, 4 h, and 7 h at 37 °C, then bacterial cells were collected with centrifugation (4500× *g* for 30 min), then washed with saline buffer, re-centrifuged, and subjected to SEM preparation (including fixation with 2% paraformaldehyde fixative solution in 0.1 M of Na-Cacodylate buffer then treatment with 2.5% of glutaraldehyde for 30 min at pH 7.3, washing with MQW and dehydration with series of ethanol concentrations). Dehydrated specimens were fixed onto SEM stubs and covered using gold/palladium, then micrographs were captured.

### 2.5. Treatment of Shrimp with UFD-ZnONPs

#### 2.5.1. Application on Peeled Shrimp

After manual deshelling, deheading, and cleansing with dDW of freshly harvested shrimps (*F. indicus*), they were categorized into five groups (consisted each of 15 pieces with ~10 ± 1 *g* weight/shrimp). First group was untreated (control, C), the second group was immersed into *U. fasciata* extract and the other three groups of shrimp were immersed into UFD-ZnONPs solutions with the following order: Groups UFD-ZnONPs 1, UFD-ZnONPs 3 and UFD-ZnONPs 5, which had with 1%, 3% and 5% (*w*/*v*) of UFD-ZnONPs, respectively. The shrimp’s treatment was executed via dipping of shrimp samples for 30–35 sec in UFD-ZnONPs solutions, at shrimp/UFD-ZnONPs solution ratio of 1:2 (*w*/*v*), followed by drainage at 25 ± 2 °C for 5 min. Samples were held at 4 ± 1 °C for 6 days and inspected each 2 days [20,21].

#### 2.5.2. Microbiological Investigation

The UFD-ZnONPs—Treated and controlled shrimps were analyzed aseptically (15 *g*/sample), soaked in 135 mL of buffered peptone (0.1 per cent, LAB M, Lancashire, UK) in a stomach sac and then homogenized for 3 min in Seward Stomacher 400 (Norfolk, UK). Serially diluted shrimp homogenates were made with NB and screened for the counts of different microbiological groups thru plating onto suitable agar media demonstrated by the standardized microbiological protocols:

[ISO 4833-1: (2013)]: “Total aerobic microorganisms enumeration of–colony count at 30 °C” [22].
[ISO 16649-1: (2018)]: “Enumeration of *Escherichia coli* (β-glucuronidase-positive)” [23].
[ISO 21528-2: (2017)]: “Enterobacteriaceae detection and enumeration” [24].
[6888-1: (2018)]: “Coagulase-positive staphylococci enumeration” [25].

#### 2.5.3. Sensorial Analysis

A well-qualified panelist group (13 members; five males and eight females), experienced with seafood assessment, was integrated in assessing the sensorial qualities of UFD-ZnONPs-treated shrimps. It was confirmed that “All members gave their informed consent for inclusion before they participated in the study”. Panelists were queried to judge samples′ color, odor, texture and appearance, using a ranged scale from 9 (extremely good) to 1 (extremely poor) [26].

## 3. Results and Discussion

### 3.1. Phycosynthesis of UFD-ZnONPs

Initially, the Zn(CH_3_CO_2_)_2_·2H_2_O solution was colorless. After addition of *U. fasciata* cell-free extract, the reaction mixture possessed a pale green color. After 4 h, the color of the mixture turned pale white indicating UFD-ZnONPs formation. These observations were coordinated with those mentioned by Ishwarya et al. [17].

**Figure 1:**
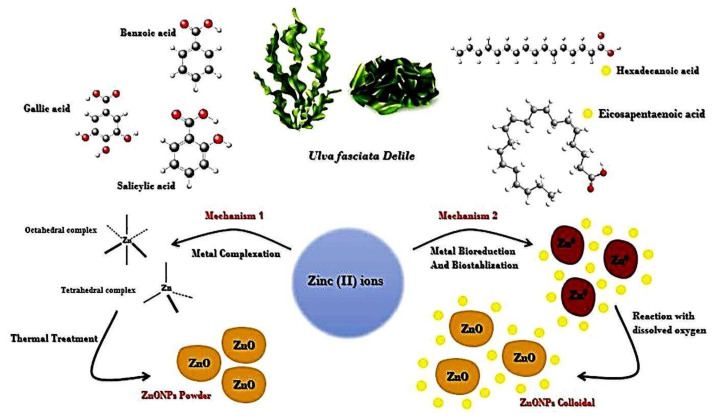
Possible mechanisms of *ZnONPs* phycosynthesis by *Ulva fasciata Delile* macroalgae.

Regarding phycosynthesis of ZnONPs, there are two common mechanisms that were commonly adopted by researchers, wherein the first mechanism suggests that the biomolecules of the algal extract chelate the zinc ions (Zn^2+^) to form complexes that are further calcinated to degrade such complexes and form ZnONPs [27,28,29]. Differently, the second mechanism proposes that zinc ions (Zn^2+^) are reduced by the algal compounds to zinc metal (Zn^0^) which reacts with presented dissolved oxygen in solution for developing ZnO nuclei. Furthermore, the algal compounds (e.g., protein and fatty acid molecules) act as stabilizers that prevent NP agglomeration (Figure 1) [30].

### 3.2. Characterization of the Phycosynthesized UFD-ZnONPs

The characterizations of extracted/synthesized agent (*U. fasciata* extract and UFD-ZnONPs) were based on the dried powders of the agents, whereas the plain ZnONPs were characterized after composite calcination.

#### 3.2.1. FTIR Analysis

**Figure 2:**
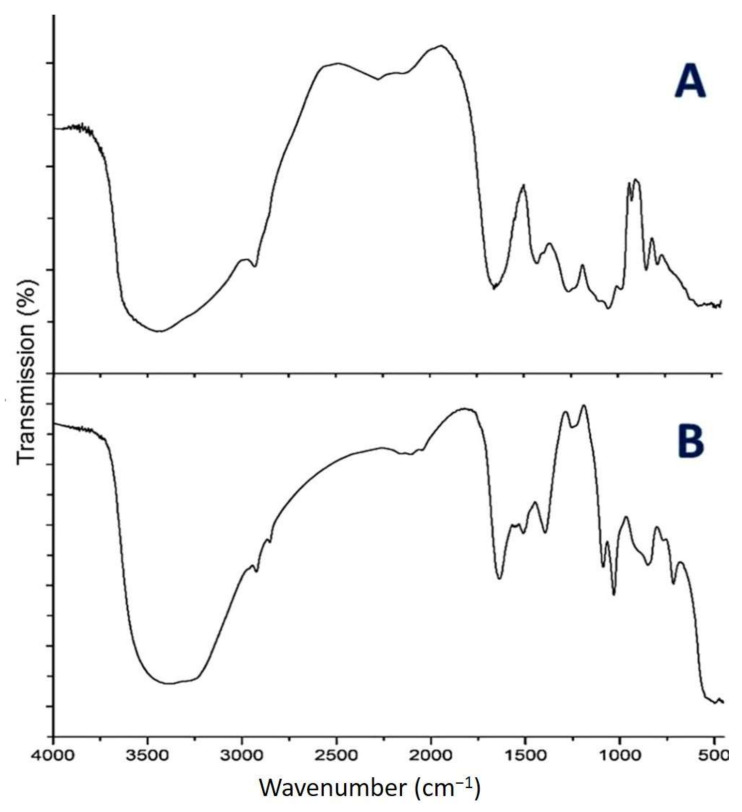
FTIR spectra of *U. fasciata* extract (**A**) and phycosynthesized *ZnONPs* with the extract (**B**).

According to Figure 2A, the cell-free extract of *U. fasciata* showed strong transmission peaks at 3441 cm^−1^, 2930 cm^−1^, 1660 cm^−1^, 1450 cm^−1^, 1250 cm^−1^, and 1020 cm^−1^. The peak at 3441 cm^−1^ was indicator to vibrated O–H stretching in presented phenols. The correspondent peak to vibrated C–H stretching of alkenes was detected at 2930 cm^−1^. The sharp peak at 1660 cm^−1^ was due to the vibrated C=O stretching of amides. The bands at around 1450 cm^−1^ are attributed to C–C stretch of aromatics. Moreover, the vibrated C–N stretching of aliphatic amines could be indicated from peaks at 1250 cm^−1^ and 1020 cm^−1^. Similar results were obtained by Radhika and Mohaideen [31].

To insure that phycosynthesis took place, referring to Figure 2B, phycosynthesized ZnONPs is obviously surrounded by phytochemicals from *U. fasciata*. Clearly, the broad intense peak at 3441 cm^−1^ of cell-free extract of *U. fasciata* was shifted to 3352 cm^−1^ in ZnONPs, which suggested that zinc has interacted with the hydroxyl group of phenolic compounds and facilitated the phycosynthesis. Obviously, the peaks at 2930 cm^−1^, 1660 cm^−1^, 1450 cm^−1^, 1250 cm^−1^, and 1020 cm^−1^, which respectively correspond to alkenes, amides, aromatics, and aliphatic amines of the *U. fasciata* extract, were shifted in the spectrum of UFD-ZnONPs (Figure 2B), which imply that these groups has enabled the phycosynthesis of ZnONPs. In general, metals’ oxides were distinguished from absorption bands below 1000 cm^−1^ (due to interatomic vibrations) [32]. The ZnONPs stretching were found around 400–700 cm^−1^ [33].

#### 3.2.2. XRD Investigation

**Figure 3:**
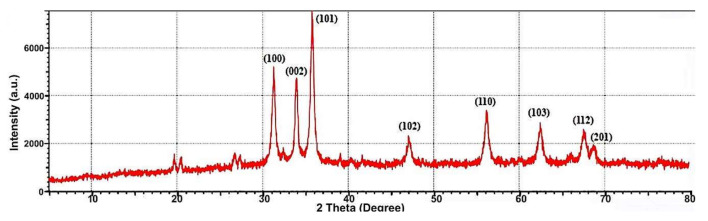
XRD Spectrum of phycosynthesized *ZnONPs*.

The plotted XRD pattern revealed the formation of crystalline phycosynthesized ZnONPs. The spectrum appointed sharp diffraction peaks (DP) of XRD at 31.2° (100), 34.4724° (002), 36.322° (101), 47.6097° (102), 56.6208° (110), 62.9286° (103), 68.0033° (112), 69.1416° (201), respectively (Figure 3). The results of pure ZnONPs matched the JCPDS (Joint Committee on Powder Diffraction Standard), file number 36-1451. This validates the ZnONPs hexagonal wurtzite structure. The detected sharp peaks indicate ZnONPs’ good crystallinity degree. Nonappearance of further prominent DP, other than ZnONPs attributed peak s, indicates the high purity degree of phycosynthesized ZnONPs.

#### 3.2.3. EDX Analysis

**Figure 4:**
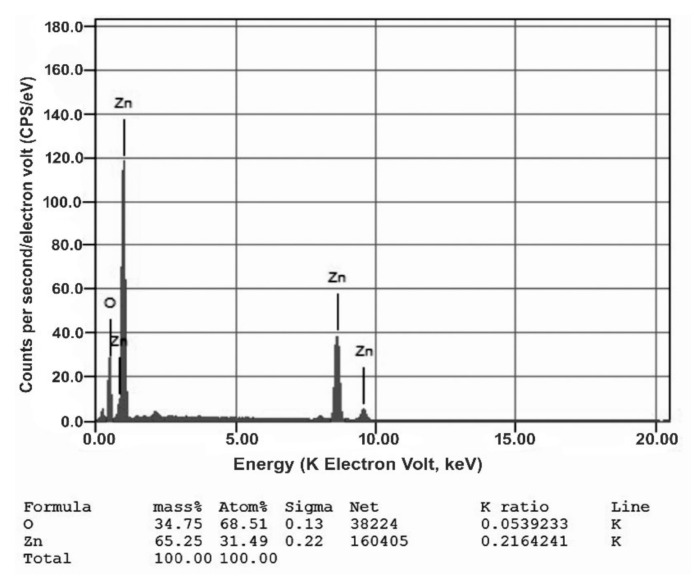
EDX spectrum of phycosynthesized *ZnONPs*.

Figure 4 shows the EDX of phycosynthesized ZnONPs, after their calcination, which reveals the presence of 65.25% Zn and 34.75% O, which indicates the phycosynthesis of pure ZnONPs. The purified nanoparticle exhibits the promising antibacterial potentiality.

#### 3.2.4. SEM Analysis

**Figure 5:**
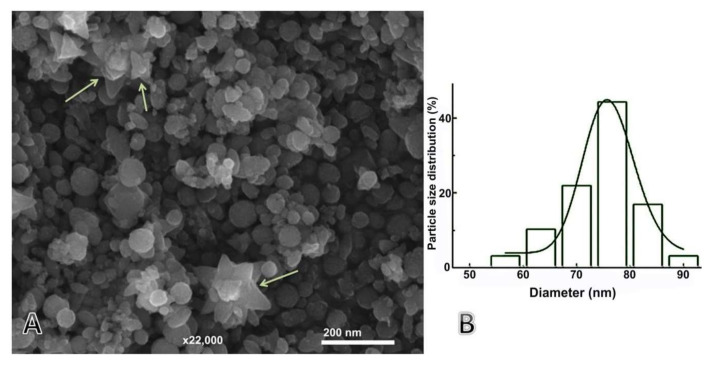
SEM micrographs (**A**) and size distribution (**B**) of phycosynthesized *ZnONPs*.

The SEM image of phycosynthesized ZnONPs reveals the shapes of ZnONPs were spheres and some of NPs had flower shapes (Figure 5). The nanoflowers were previously reported to be formed when using Zn(CH_3_CO_2_)_2_·2H_2_O as a precursor, whereas self-assembled nanorods formed porous leaf-like structures; thus, many of these structures could eventually form the larger nanoflowers [32,33,34]. In Figure 5A, a few leaf-like structures (marked by arrows) were observed, which, as previously mentioned, supported the building of mechanistic pathways to form ZnO nanoflowers. Thus, conjoining of many leaf-like forms will eventually lead to ZnO nanoflower structure formation [34]. The algal compounds can perform imperative roles in stabilizing these nanostructures [35,36].

#### 3.2.5. Particle Size (Ps) Distribution and Zeta Potential (ζ) Analysis

Typically, NPs with high values of zeta potential (ζ) greater than +30 mV or less than −30 mV exhibit higher stability degrees because of the inter-particle electrostatic repulsion [37]. The photosynthesized calcinated ZnONPs were confirmed to be positively charged with ζ values of +18.6 mV, which indicates moderate stability of the nanoparticles with a moderate tendency of the particles to agglomerate. The detected Ps of ZnONPs exhibits the Ps distribution from 53 nm to 96 nm with calculated mean and median Ps of 78 nm and 75 nm, respectively (Figure 5B).

### 3.3. Evaluation of Antibacterial Potentiality

#### 3.3.1. Qualitative Antibacterial Potentiality: Iz Assay

The antibacterial potentiality of the cell-free extract of *U. fasciata* (UFD) and phycosynthesized UFD-ZnONPs was investigated toward two bacterial pathogens strains, *E. coli* and *S. aureus*. The results indicate that the highest antibacterial potentiality against both strains was for UFD-ZnONPs, where *E. coli* was found to be more sensitive than *S. aureus* (Table 1). The antibacterial potentiality of NPs can be attributed to the extra surface area available that interact with cellular membranes of the bacteria and, accordingly, have extreme interaction with microbes. This interaction may lead to the increase in penetrability into the exterior membrane leading to NP entry inside the cells and affecting the cellular viability [38]. However, UFD-ZnONPs were more potent than pure ZnONPs, which could be explained by the existence of phenols, amino acids, and fatty acids of the UFD extract. Moreover, UFD, alone, showed no IZ toward both strains; this can refer to the poor interaction of the extract with cellular wall of Gram-positive, and the impermeability of the lipopolysaccharides barrier and teichoic acids absence in Gram-negative cell walls [39]. These observations were in line with those performed on Gram-positive and Gram-negative strains [40].

#### 3.3.2. Quantitative Antibacterial Potentiality: MIC

After challenging the bacteria with of 1–200 µg/mL serial concentrations of ZnONPs, the results indicate that UFD-ZnONPs showed a smaller MIC values than pure ZnONPs (Table 1). This demonstrates that UFD-ZnONPs that possessed an extra potential of UD capping agents and bioactive compounds can inhibit bacteria at a lower MIC, whereas pure ZnONPs can inhibit bacteria at a higher concentration for both strains. These observations were in line with those mentioned by Elumalai and Velmurugan [41].

#### 3.3.3. SEM Imaging

**Figure 6:**
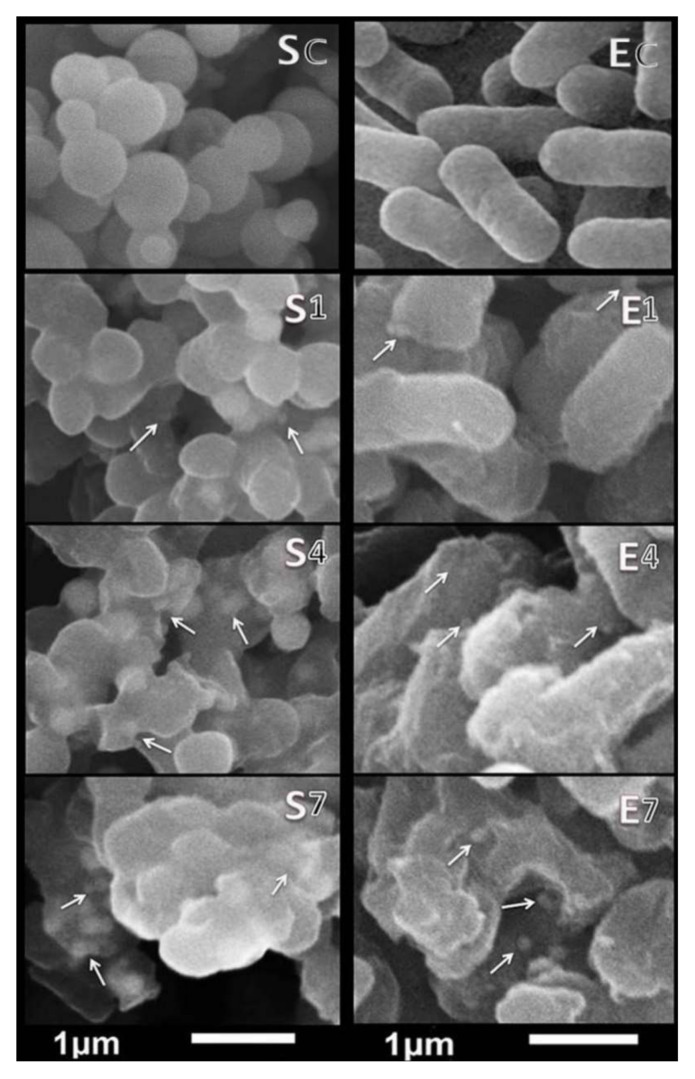
Scanning micrographs of treated *Staphylococcus aureus* (left) and *Escherichia coli* (right) with phycosynthesized UFD-ZnONPs after 1 h (**S1**,**E1**), 4 h (**S4**,**E4**), and 7 h (**S7**,**E7**), compared with non-exposed bacteria (**SC**,**EC**). * Arrows indicate some of attached NPs to cell matrix.

The non-exposed bacteria (Figure 6SC,EC) appeared with uniform, contacted, and smooth outlines, indicating their healthy status. After 1 h of treatment (Figure 6S1,E1), many control (normal) cells were detected, with smooth and contacted walls wherein some phycosynthesized UFD-ZnONPs began to attach to cells and interact with them. The SEM manifested that the treated bacterial cell membranes of *S. aureus* and *E. coli* had been deformed and disorganized after 4 h of exposure to ZnONPs (45 µg/mL). Many *E. coli* cells were lysed, whereas some of *S. aureus* began to deform with more accumulation of UFD-ZnONPs in the membranes (Figure 6S4,E4). After 7 h of exposure, most of the treated cells from *S. aureus* and *E. coli* were exploded/lysed and the few intact cells that remained were viewed in amalgams with leaked internal constituents (Figure 6S7,E7).

**Figure 7:**
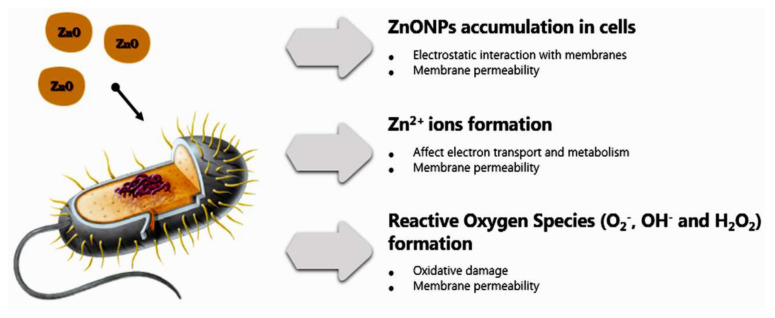
Possible mechanisms of *ZnONPs* antibacterial potentiality.

The phycosynthesized UFD-ZnONPs are positively charged and, thus, showed a quickly severe damage to *E. coli* more than *S. aureus*. However, the positively-charged UFD-ZnONPs may also interact with negatively-charged teichoic acid component in *S. aureus*. Additionally, zinc ions may affect the system of electron transportation and increase the generation of ROS (reactive oxygen species) [42]. Such interactions will cause membrane damage, cytoplasm leakage, and eventually cell death (Figure 7). The different *ZnONPs* shapes, e.g., nanoparticles, nanorods, nanowires, and nanoflowers, were entirely reported to possess elevated microbicidal potentialities toward various pathogens [40,41,42,43,44,45]; the nanoflower shape was supposed to have higher potentiality for antimicrobial applications [44].

### 3.4. Treatment of Shrimp with UFD-ZnONPs Solution

#### 3.4.1. Microbiological Examination

**Figure 8:**
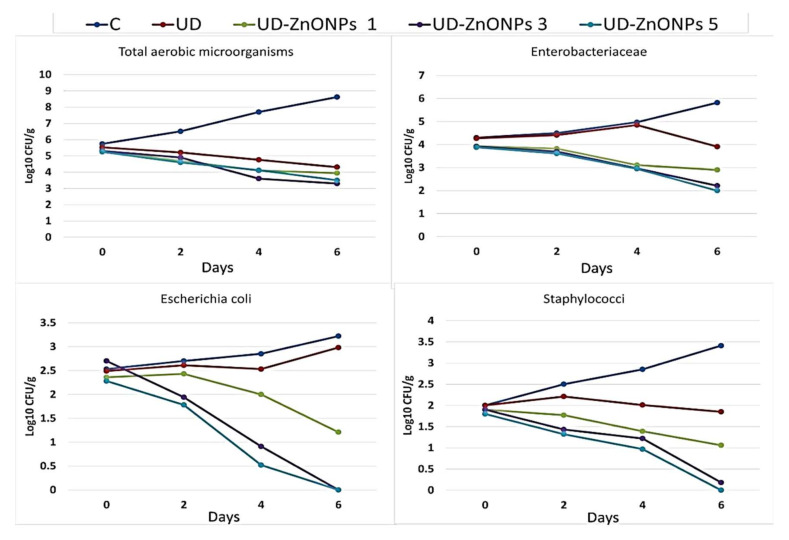
Influence of shrimp treatment with UFD extract and 1, 3 and 5% (*w*/*v*) from *UFD-ZnONPs* (*UFD-ZnONPs* 1, *UFD-ZnONPs* 3, and *UFD-ZnONPs* 5, respectively) on the microbial load during six days of storage (4 ± 1 °C), compared to non-*UFD-ZnONPs* treated group (C).

The influences of shrimps’ treatment with UFD-ZnONPs, at of 1, 3, and 5% ratios (*w*/*v*), on the survival of microbial groups throughout storage under refrigeration (4 ± 1 °C), are exemplified (Figure 8). Whereas the number of entire inspected microbial groups (*E. coli*, Enterobacteriaceae, Aerobic microorganisms, and coagulase + staphylococci) sharply increased, in untreated (control) groups, with storage extension, the UFD-ZnONPs treatments exhibited remarkable inhibitory actions toward all microbial groups.

Significantly, the UFD-ZnONPs preparation reduced the microbiological loads in preserved shrimps, compared with untreated samples. The antimicrobial capability generally increased with UFD-ZnONPs increment; the most powerful concentration from UFD-ZnONPs was 5% *w*/*v*. This formulation could decrease the cells′ counts of *E. coli* and coagulase + staphylococci to zero after cold storage for four days. The UFD-ZnONPs treatment was evidently effective for hindering bacterial growth; its efficacy particularly augmented with increasing UFD-ZnONPs concentration.

This antimicrobial potentiality was attributed to its accumulation in cells, release of Zn^2+^ ions, generation of ROS and their destructive interactions with interior microbial components (Figure 7). Another factor mentioned by researchers was the high photocatalytic efficiency which significantly activates interactions of ZnONPs with bacteria. Additionally, morphology can positively affect antimicrobial potential, as flower-shaped can possess higher microbicidal actions toward *E. coli* and *S. aureus*, than the rod- and spherical-shaped *ZnONPs* [34,35,36,44]. Moreover, the antibacterial efficiency depends on the Ps of ZnONPs. Smaller Ps could effortlessly penetrate to bacterial membranes because of their great interfacial capacity and, accordingly, augment their bactericidal efficiency [45].

The antimicrobial consequences generated from *Ulva* species extracts were frequently demonstrated toward numerous species from pathogenic microorganisms, especially foodborne species [46]. Specifically, microbicidal impacts of *U. fasciata* are attributed to the phenolic compounds, especially benzoic acid and gallic acid, in addition to fatty acids and nonpolar compounds. This confirmed the ability of extract to have antimicrobial activity due to the existence of a methoxy group (OCH_3_) in a benzene ring at the meta position, carboxylic group (COOH), and two hydroxyl (OH) groups in ortho/para positions appeared to be essential [28]. The *U. fasciata* extract definitely empowered ZnONPs, leading to a combined synergistic antimicrobial effect which was persevered throughout storage extent of peeled shrimps.

#### 3.4.2. Sensory Analysis

**Figure 9:**
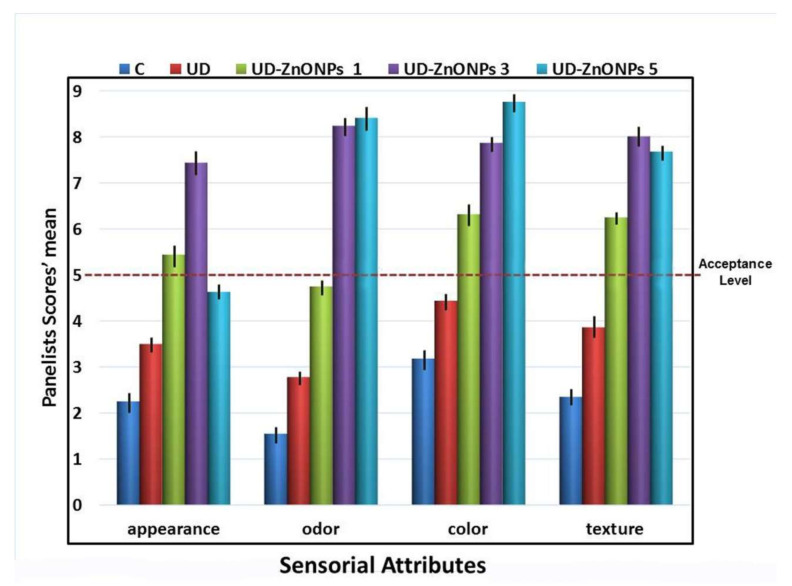
Impact of shrimp treatment with UFD extract and 1, 3 and 5% (*w*/*v*) from UFD-ZnONPs (UFD-ZnONPs 1, UFD-ZnONPs 3, and UFD-ZnONPs 5, respectively) on sensorial attributes* after six days of storage (4 ± 1 °C), compared to non-UFD-ZnONPs treated group (C)**. * The scale (1−9) indicates the range from extremely poor (1) to extremely good (9). ** Vertical bars on column tops indicate the standard deviation.

The influence of shrimp treatment with UFD extract and UFD-ZnONPs (at concentrations of 1, 3, and 5%), on sensorial characteristics after cooled storage for six days (4 ± 1 °C), is obtainable from Figure 9. While no remarkable differences were detected for the examined sensorial characteristics in all samples in the experiment beginning (data not included), the panelists team’s scores for these attributes (appearance, color texture, and odor) showed that control (untreated) sample and UD-treated sample were inconsumable by this time, concerning the “acceptance level” of 5/9 as the restrictive value. Significantly, all sensorial scores for UD-treated and control groups were much lesser than UFD-ZnONPs-treated samples. The best concentration of UFD-ZnONPs, to uphold texture and appearance of stored shrimps, was 3%, whereas the best concentration for odor and attribute was 5%.

**Figure 10:**
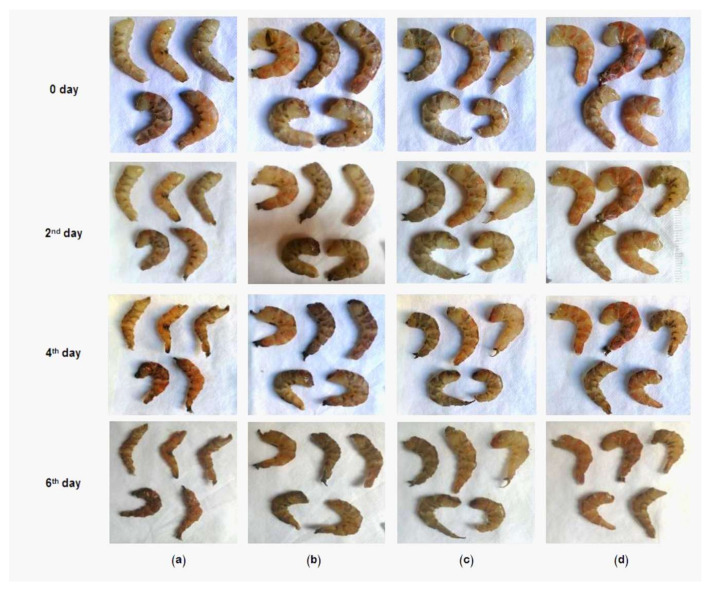
Appearance and texture changes of shrimps stored at 4 °C for 0, 2, 4, and 6 days when treated with 1% UFD-ZnONPs (**b**); 3% UFD-ZnONPs (**c**); and 5% UFD-ZnONPs (**d**) compared to control (**a**).

The upholding of texture, color and appearance qualities, in UFD-ZnONPs-treated shrimps, was remarkably demonstrated, compared to control samples (Figure 10). Numerous healthiness profits from seafoods are mainly accredited to their high contents of valuable lipids, specifically omega-3 besides long-chained PUFA “poly unsaturated fatty acids” [47]. These cherished components are unfortunately susceptible to fast oxidation, which develops the off-flavors, throughout most storage conditions. The UFD-ZnONPs application for shrimp’s biopreservation could maintain the sensorial quality via the shield from oxidation. ZnONPs was stated as influential water vapor barriers, ultraviolet-blocking agents, lipid oxidation retarders and oxygen barriers [48]. The oxygen barrier and ultraviolet-blocking properties of UFD-ZnONPs protected peeled shrimps from oxidative reactions that frequently occurred. It has been reported that increasing the concentration of ZnONPs will decrease ultraviolet transmittance, which is one of the factors accelerating lipid oxidation [49]. Moreover, increasing concentration of ZnONPs was assumed for increasing water vapor permeability, which, in turn, positively affects the sensory attributes of peeled shrimps [50].

The sensorial quality maintenance, in UFD-ZnONPs treated shrimps, is evidently associated with the lowered microbial burden. Numerous findings suggested ZnONPs application and their nanocomposites with other bioactive organic and inorganic ingredients for meat, fruits, and vegetables preservation, whereas shrimp and seafood were investigated in a lesser extent [51,52].

The diverse ZnO nanoforms (nanoparticles, nanowires, nanoflower, etc.) were considered as promising candidates to inhibit foodborne microbial pathogens in food systems, regarding high biocobatibility, biosafety, and environment-friendly natures of these nanoforms [9,16,33,53]; they suggested that closely-applied concentrations to 100 μg/mL of nano-ZnO could be compatible and safe to cellular activities; this biosafety is assumingly increased by the nanoparticles combination with biological matters (e.g., *U. fasciata* extract) before applications. The achieved results here were with those mentioned by Baek and Song [54], which showed that *Gracilaria vermiculophylla* extract nanocomposite comprising 3% ZnONPs exhibited strong antibacterial actions for bacterial pathogens and reduced degrees from lipid oxidation, recommending that these nanocomposites could be effectually employed as active materials for food (smoked salmon) packaging. On the other hand, Indumathi et al. [55] constructed biodegradable films comprising chitosan/CAP (cellulose acetate phthalate) and contained 5% from ZnONPs, which exhibited the ideal level of thermal, mechanical, and UV-protective characteristics, besides their strong microbicidal actions against *E. coli* and *S. aureus*, which prolonged grapefruit shelf-life for nine days. Moreover, incorporating 2% ZnONPs in BSG (bovine skin gelatin) and cloves oil film improved oxygen and UV barrier property and achieved the highest bactericidal actions against inoculated *S. typhimurium* and *Listeria monocytogenes* in shrimp throughout cooled storage [56].

## 4. Conclusions

Phycosynthesis of UFD-ZnONPs from the *U. fasciata* cell-free extract could be introduced as a simple, cost-effective, eco-friendly nanobiotechnological method. The phycosynthesis of UFD-ZnONPs were achieved using Zn(CH_3_CO_2_)_2_·2H_2_O to give UFD-ZnONPs a mean size of 77.81 nm, with flower and sphere shapes and positive zeta potential. Phenolics, fatty acids, and amino acids in the *Ulva* extract served as capping and reducing molecules on these UFD-ZnONPs. These phycosynthesized UFD-ZnONPs manifested potent anti-bacterial potentiality against foodborne pathogenic Gram-positive and Gram-negative bacterial strains. The scanning micrographs of treated *E. coli* and *S. aureus* cells indicated that antibacterial action also depended on the time of exposure of bacterial cells to UFD-ZnONPs. Peeled shrimps treated with 3% and 5% UFD-ZnONP nanocomposites displayed improved sensorial qualities and decreased microbial profile, respectively, compared to untreated shrimps during 6 days of storage at 4 °C.

## Figures and Tables

**Table 1:** Antimicrobial potentialities of phycosynthesized annealed zinc oxide nanoparticles (*ZnONPs*), *Ulva fasciata Delile* extract (UFD extract) and their nanocomposite (*UFD-ZnONPs*) against bacterial pathogens.

Examined Nanoparticles	Antibacterial Potentiality			
	*E. coli*		*Staphylococcus aureus*	
	ZOI (mm) *	MIC (µg/mL)	ZOI (mm)	MIC (µg/mL)
UFD extract	ND	200	ND	175
ZnONPs	23.4 ± 1.6	27.5	19.7 ± 1.1	22.5
UFD-ZnONPs	27.4 ± 1.3	25	24.9 ± 1.5	17.5

* ZOI “Inhibition zones” represent triplicates means ± SD (standard deviation), comprising 6 mm diameter of disc assay that carried 100 µg of nanoparticles.
